# Glycemic Index Values of Pasta Products: An Overview

**DOI:** 10.3390/foods10112541

**Published:** 2021-10-22

**Authors:** Giuseppe Di Pede, Rossella Dodi, Cecilia Scarpa, Furio Brighenti, Margherita Dall’Asta, Francesca Scazzina

**Affiliations:** 1 Department of Food and Drug, University of Parma, Via Volturno 39, 43124 Parma, Italy; giuseppe.dipede@unipr.it (G.D.P.); rossella.dodi@unipr.it (R.D.); cecilia.scarpa@studenti.unipr.it (C.S.); furio.brighenti@unipr.it (F.B.); 2 Department of Animal Science, Food and Nutrition (DiANA), Università Cattolica del Sacro Cuore, Via Parmense 84, 29122 Piacenza, Italy; margherita.dallasta@unicatt.it

**Keywords:** pasta, glycemic index, enrichment, carbohydrate, ingredient, database, formulation, quality

## Abstract

Durum wheat pasta is considered a low-glycemic index (GI) food. In recent years, the interest in developing enriched pasta has increased. Since both the formulation and processing technologies may affect the GI, this study aimed to investigate the GI values of pasta products (pp) reported in the literature until 2020. GI values of pp analyzed following the ISO guidelines were included in this survey. A total of 95 pp were identified and, according to their formulation, classified into 10 categories (n, mean GI): category n 1: 100% refined wheat (35, 55); category n 2: 100% whole wheat (6, 52); category n 3: other cereal-based products (8, 52); category n 4: containing egg (5, 52); category n 5: gluten free (11, 60); category n 6: containing legumes (9, 46); category n 7: noodles and vermicelli (9, 56); category n 8: containing vegetable or algae (6, 51); category n 9: containing other ingredients (5, 37); category n 10: stuffed (1, 58). Overall, pasta is confirmed to be a medium–low-GI food, even if a high variability among or within each category emerged. The formulation of enriched pp able to elicit a controlled glycemic response could represent a strategy to improve the nutritional value of pasta.

## 1. Introduction

**Figure 1:**
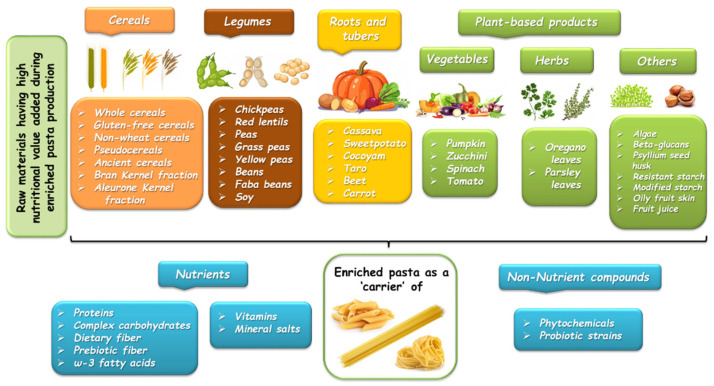
Raw materials commonly employed to produce enriched pasta product at high nutritional value.

Cereals, tubers and pulses are the main dietary sources of carbohydrates within the human diet [1], which are well known as the main dietary components affecting postprandial blood glucose levels [2,3,4]. The glycemic index (GI), proposed by Jenkins [5], is a tool for quantifying the relative rise in blood glucose level after consuming a carbohydrate-containing food. The GI is defined as the incremental area under the two-hour blood glucose response curve (IAUC) after ingestion of a food with a certain amount of available carbohydrates, expressed as a percentage of the IAUC after consumption of a standard meal in an iso-glucidic portion [5,6]. Pasta, a traditional food item within the Italian diet, is now globally consumed, becoming an important source of complex carbohydrates (i.e., starch) in many countries [7,8]. Since durum wheat pasta is produced by mixing semolina with water and with energy input [9], its nutritional properties are prevalently linked to its matrix structure formed during the extrusion and drying processes [10,11,12]. As a consequence of this technological process, the microstructure of pasta is compact and relatively dense, limiting the hydrolysis of internal starch granules, which explains its richness in slow digestible starch and its reduced enzymatic susceptibility during digestion [9,12]. Postprandial studies conducted in both healthy and diabetic volunteers confirmed that durum wheat pasta induced a lower postprandial glucose response than other wheat-based products (i.e., bread) by virtue of its compact dense physical structure (dried pasta) and the network of gluten surrounding the starch granules [13,14,15,16]. On the other hand, refined wheat pasta is significantly lower in fiber and micronutrients (i.e., minerals and vitamins) with respect to whole grain pasta [9], and it is well known that the biological value of wheat proteins is low due to the deficiency in some essential amino acids, such as lysine and threonine [17]. Due to the importance and role of pasta as one of the main staple foods in the human diet, the interest in developing enriched pasta with high nutritional values has grown [18,19,20,21,22]. To achieve this goal, different approaches have been developed, as pasta could be used as dietary carrier of macronutrients, vitamins, minerals and/or phytochemicals by adding legumes, flour from vegetables/marine foods, and flour of refined or whole cereals different from wheat [19,20,23,24,25] (Figure 1).

Within the context of enriched pasta, functional ingredients can be added, as functional food consumption has increased in recent years [26,27]. Their consumption, by virtue of their physiologically active components, should provide health benefits beyond basic nutrition [28]. Since pasta formulation could affect the glycemic response after consumption, and therefore, its GI, beyond the processing method [29,30,31], a large number of human intervention studies have investigated the GI of enriched pasta products [18,21,32,33,34]. Thus, since the GI represents one of the most important parameters considered for evaluating the quality of dietary carbohydrates, this study aimed to gather the GI values of pasta products (pp) published in the literature until 2020.

## 2. Materials and Methods

### 2.1. Data Collection

A literature search to collect data on GI values of pp published in the literature without any restrictions was performed in December 2020 by using Pubmed, Scopus, Web of Science and Science Direct. Keywords used for data collection were: “glyc(a)emic index” AND pasta. Taking into consideration the ISO guidelines [35] for GI determination, exclusion criteria for data collection were as follows: (I) GI values obtained in the context of mixed meals or with the addition of any condiments; (II) GI values obtained using a sample size of less than ten subjects, and/or unhealthy subjects; (III) GI values calculated by using a standard meal other than glucose solution or white bread; (IV) GI values calculated considering IAUCs obtained before or after two postprandial hours following pasta consumption; (V) human intervention studies not specifying the number of subjects enrolled and/or the standard meal used; (VI) GI values calculated using in vitro models (i.e., estimated GI).

### 2.2. Database Development

Data on (i) pasta characteristics (types and formulation), (ii) GI values (mean value and data distribution expressed as standard deviation (SD) or standard error of the mean (SEM)), and (iii) experimental protocol for GI measurement (blood sample, sample size, standard meal, available carbohydrate (Av. CHO)/portion in grams, and place of analysis) were collected from research papers that met the inclusion criteria. According to their formulation, pp were classified into ten categories: (category n 1) 100% refined wheat; (category n 2) 100% whole wheat; (category n 3) other cereal-based products; (category n 4) containing egg; (category n 5) gluten free (GF); (category n 6) containing legumes; (category n 7) noodles and vermicelli; (category n 8) containing vegetable or algae; (category n 9) containing other ingredients; (category n 10) stuffed. Furthermore, pp within the same category were further subdivided into ‘Low’ GI (0 ≥ GI ≤ 55), ‘Medium’ GI (55 > GI ≤ 70), and ‘High’ GI (70 > GI ≤ 100) [5,35].

### 2.3. Data Analysis

The normality of data distribution within each category was verified through the Kolmogorov–Smirnov test, and GI values for the 10 categories of pp were expressed as the mean. The number of items at low, medium and high GI was provided as a percentage value with respect to the total number of pp within each category (data distribution). The statistical analysis was carried out using SPSS software (IBM SPSS Statistics, Version 25.0, IBM Corp., Chicago, IL, USA).

## 3. Results

### 3.1. GI Data

**Figure 2:**
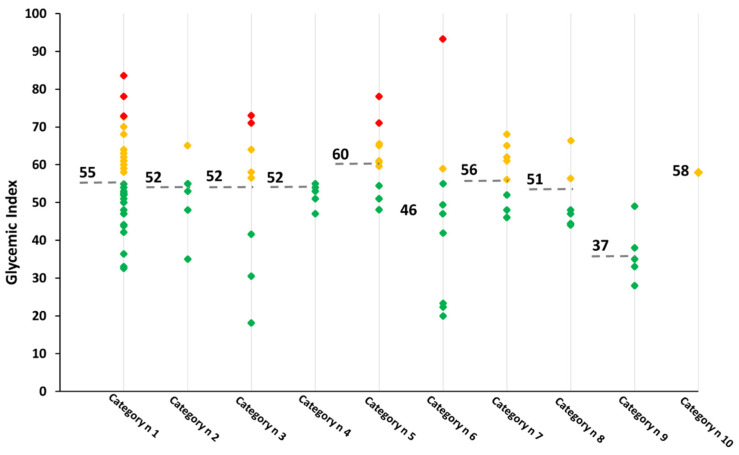
GI values of the 10 categories of pp analyzed. Red diamonds correspond to high GI pp; orange diamonds to medium GI pp; green diamonds to low-GI pp. Values reported in the figure correspond to the mean value for each category. Note: category n 1: 100% refined wheat; category n 2: 100% whole wheat; category n 3: other cereal-based products; category n 4: containing egg; category n 5: gluten free; category n 6: containing legumes; category n 7: noodles and vermicelli; category n 8: containing vegetable or algae; category n 9: containing other ingredients; category n 10: stuffed.

GI values of 95 pp were gathered from 28 research articles and are reported in Table 1. Category n 1 (100% refined wheat) was the largest group, including 35 items, among which six values were collected for 100% whole wheat pasta (category n 2), eight for other cereal-based products (category n 3), five for egg pasta (category n 4), 11 for GF (category n 5), nine for products containing legume (category n 6), nine for noodles and vermicelli (category n 7), six for pasta containing vegetable or algae (category n 8), five for items containing other ingredients (category n 9), and only one for stuffed pasta (category n 10). As reported in Figure 2, the GI of pp belonging to the same category are highly variable. Low-GI pastas were present in all the investigated categories, with the only exception of category n 10 (stuffed pp). No data on medium GI food items were recovered for products containing egg and containing other ingredients (categories n 4 and n 9, respectively). Conversely, high GI pastas fell within the 100% refined wheat pasta (category n 1), other cereal-based products (category n 3), GF pasta (category n 5) categories, and within products containing legumes (category n 6).

According to the GI classification rank (http://www.glycemicindex.com, accessed on 20 July 2021), pp belonging to categories n 1 (100% refined wheat), n 2 (100% whole wheat), n 3 (other cereal-based products), n 4 (containing egg), n 6 (containing legumes), n 8 (containing vegetable or algae), and n 9 (containing other ingredient) can be classified as low-GI foods. Items belonging to categories n 5 (gluten free), n 7 (noodles and vermicelli) and n 10 (stuffed) had a medium GI.

### 3.2. Formulations

Flours from barley and emmer were the main flours employed to produce pp with other cereals (category n 3), followed by spelt and Kamut^®^ flours. GF items (category n 5) were formulated using GF cereal flours (rice, corn, and millet) and adding legumes (chickpea, soy), or modified starches (high amylose or resistant maltodextrin). Among the items containing legumes (category n 6), only three were formulated with 100% legume flour (red lentil, pea, and soy), while the remaining products were produced through a combination of legume (faba bean, chickpea, and whole yellow pea) and durum wheat flour, or by mixing different legume flours (i.e., grass pea and chickpea flours). Flours from wheat, rice, corn, or tubers (i.e., tapioca) were raw materials used for the formulation of noodles and vermicelli (category n 7). Pulps from carrot, pumpkin, tomato, zucchini and spinach were used for pasta containing vegetable formulations, while only one algae flour type (Eucheuma cottonii), added at different percentages (7%, 14%, and 21%), was used for pasta containing algae production (category n 8). Items containing protein, starchy ingredients (amylose and resistant starch) or fiber (Barley Balance^®^, psyllium seed husk) were included in category n 9 (containing other ingredients).

### 3.3. Experimental Protocol Data

A total of 71 GI values (equal to 75% of the total GI values) were obtained from capillary blood with respect to venous blood (used for 4% of the total GI values), and in the remaining studies, this information was not available. A total of 74 GI values (78% of the total GI values) were calculated with a sample size of 10 subjects. Glucose solution as a standard meal was used for the determination of 76 GI values (80% of the total GI values). For 73 GI values, the amount of available carbohydrates (Av. CHO) contained for each portion of pasta was 50.0 g, while for 16 GI values, the Av. CHO content in pasta portion size ranged from 22.0 g to 49.0 g; no data were available for the six remaining products. Italy was the place of analyses for 42 GI values (equal to 44% of the total GI values), while a great heterogeneity emerged for the remaining items.

## 4. Discussion

This study aimed to develop a database of GI values of pp based on the collection of the data recently reported in the literature. To the best of our knowledge, this is the first database specifically designed for reporting all GI data on pp, even if several databases on GI values, calculated either in healthy or diabetic patients, of a wide range of food items, have been proposed [37,40,45,57,58,59,60]. High-GI foods elicit higher postprandial glycemic responses, which have been associated with several chronic diseases, among which type 2 diabetes [61,62], cancer [2,63], and cardiovascular diseases [3,61] are the most relevant. Hence, since low-GI food consumption was associated with weight reduction and decreased incidence of several pathological conditions [3,4,6,61,64,65], the adherence to low-GI dietary patterns is strongly recommended by several national guidelines aiming at cardiovascular disease and diabetes prevention worldwide [66,67,68,69,70]. The present work confirmed that the GI of refined wheat pasta is low, even if a relevant variability was observed among GI values belonging to category n 1 (100% refined wheat). Indeed, among GI values gathered for category n 1, 60% of them were low (*n* = 21), followed by items of medium and high GI (29% (*n* = 10) and 11% (*n* = 4) for 100% refined wheat pastas at medium and high GI, respectively). The physical structure of the gluten matrix, formed by durum wheat starch and wheat proteins, is the main intrinsic factor supposed to explain the lower glycemic response of 100% refined wheat pasta products with respect to other products prepared with refined wheat [10,11,12,13,71]. In fact, it is well established that wheat pasta may elicit a lower postprandial glycemic response compared with bread or potatoes in both healthy and diabetic subjects [11,13,14,72,73]. The presence of high-GI pp among those belonging to category n 1 could have been probably linked to a different area of production [18,19,33,51], which reflects a certain heterogeneity in both pasta formulation and processing technology. The 100% whole wheat items (category n 2) had prevalently low GI, confirming the tendency of wheat fiber to positively modulate postprandial glycemic excursions [74]. It seems that the overall concept of the low GI of durum wheat pasta should be contextualized with the raw materials (common or durum wheat, refined or whole wheat), their origin, and the technological process used to produce it, rather than with the experimental conditions (i.e., sample size, characteristics and dietary patterns of the enrolled subjects, and inter-day variability) applied throughout the study. Despite pp belonging to category n 3 were classified as low-GI foods, it should be noted that pp formulated with whole barley flours resulted in high GI, probably due to a weaker food structure by virtue of the higher amount of insoluble fiber in whole barley [51]. Further human intervention studies are needed to fully clarify the influence of using other cereals (both in their refined and whole version) on the GI of pasta. It is well known that food formulation, as well as processing technologies, has been recognized as the most important factors affecting the GI of food products [29,30,31,75]. In the present work, enriched pp were classified into seven categories, reflecting the high variety of raw materials employed throughout the technological processing to enrich them. Nowadays, several food production/formulation strategies are implemented to enrich pasta by improving its nutritional [20,76,77,78], technological [79,80,81,82] and sensorial attributes [83,84,85,86,87]. Moreover, both nutritional and health claims could be obtained following food enrichment [88], positively affecting consumer choices [89,90,91,92]. Egg pp samples (category n 4) had a low GI by virtue of egg macronutrients, such as protein and lipids, which may mediate a reduction in the glycemic excursion [93]. Considering all the samples included in the enriched pp categories (from categories n 4 to n 10), 29 items (equal to 63% of the total enriched pp) were categorized as low GI, while the remaining 14 and 3 pp were medium and high GI, respectively (equal to 30% and 7% of the total enriched pp for those at medium and high GI, respectively). Based on these results, it is clear that enriched pasta also tends to maintain a food matrix able to make starch poorly accessible to the enzymatic activity within the gastro-intestinal tract. On the other hand, it should be considered that some raw materials added for pasta enrichment might negatively influence its GI. Among pp belonging to categories n 5 and n 7 (GF, noodles and vermicelli, respectively), a high heterogeneity in GI values for items formulated from the same starchy source (i.e., rice and corn) emerged. In this case, the absence of further details concerning both the composition and the technological processes employed for both GF and noodle and vermicelli production limits any exhaustive conclusions on the link between a product’s characteristics and its GI. Furthermore, 78% of the total legume pp (category n 6) were categorized as low-GI items. Legumes are low-GI components of the Mediterranean diet by virtue of their nutritional properties (i.e., richness in protein and low digestible starch) [94,95,96]. Similarly, 67% of pp belonging to category n 8 (containing vegetable or algae) were also low GI. If vegetables are cooked or dressed with healthful oils, they could be considered important low-GI foods within our diet [97]. On the other hand, algae are recognized for their capacity to modulate glycemic response possibly thanks to the richness in bioactive compounds able to modulate glucose absorption and disposal [98]. As reported in Table 1, it should be presumed that both soluble fiber and modified starches or protein did not affect the food matrix structure and, consequently, carbohydrate bioavailability of pastas. Indeed, 100% of the items belonging to category n 9 were low GI. Dietary fiber, hydrocolloids, resistant starches and proteins have been shown to be able to slow the carbohydrate digestion rate [99,100]. Especially for other cereal-based items (category n 3), for GF pp (category n 5), and for those containing legumes (category n 6), GI values belonging to the same category were highly variable, reflecting the putative role of food properties [29,31,101], technological processing methods [14,15,20,54,102] and cooking time [12,31,103,104] in affecting carbohydrate bioavailability for pp, which could appear similar. Furthermore, since GI data for similar pp were presented as mean values and were collected from different human studies, the possible inter-individual variability in carbohydrate metabolism should also be taken into account [31,101,105,106]. The same factors may explain the variability observed among items belonging to different categories, which were not comparable. Similar pp (i.e., in terms of type, size, and shape) have different GI, since they could have been formulated by different brands or food factories and by means of several different raw materials (i.e., non-local flours) or a variety of technological methods. This variability could be greater for foods prepared to be sold in different national markets, given that the same product could be formulated depending on the country in which it will be commercialized [19]. We collected pp without any condiment added to avoid any confounding factors, since their role in modifying the glycemic excursion was clearly demonstrated [39,107,108,109]. Finally, both data on pasta formulation or regarding the experimental protocol employed for GI measurement were not always available, representing a limitation of the present study and proving the need for well-designed studies. The lack of data for some categories limits the conclusions for a clear relation between pasta formulation and GI value.

## 5. Conclusions and Future Perspectives

Overall, pasta is confirmed to be a medium–low-GI food. The present database would be a useful tool for pasta producers to formulate enriched pp with a high nutritional value. In fact, pasta with a high nutritional value and a low GI should be the industrial target, also keeping in mind specific consumer categories (e.g., celiac disease or type 2 diabetes patients). The observed variability for GI values of pp belonging to the same category, and to different categories, proves the inevitable role of formulation in influencing the GI of pasta, one of the most consumed starchy foods in our diet. Further human intervention studies are needed to obtain a clearer picture of this relationship.

## Figures and Tables

**Table 1:** Pasta product characteristics, glycemic index and experimental protocol data.

Pasta Product Characteristics		GI Data		Experimental Protocol Data					
Types	Formulation	Mean Value	Data Distribution	Blood Sample Type	Sample Size	Standard Meal	Av. CHO (g)/Portion	Place of Analysis	Ref.
**Category n 1: 100% refined wheat**									
**Low GI**									
-spaghetti, dried at high temperature (80 °C) *	durum wheat (*var. Duilio*) flour	32.6	6.1 º	capillary	10	G	50.0	Italy	[36]
-spaghetti §	durum wheat flour	33.0	6.0 º	capillary	10	G	50.0	Italy	[37]
-spaghetti *	white wheat flour	36.4	35.8 **	venous	12	G	50.0	IS	[38]
-spaghetti *	white wheat flour	42.1	10.8 **	capillary	10	G	50.0	IS	[38]
-spaghetti *	white wheat flour	43.8	9.2 **	capillary	10	G	50.0	IS	[38]
-spaghetti *	durum wheat flour	44.0	7.0 º	capillary	13	G	50.0	Italy	[39]
-spaghetti * (CT: 15 min)	white flour	44.0	3.0 º	n.a.	10	G	48.0	Australia	[40]
-spaghetti *	white wheat flour	44.1	19.8 **	capillary	10	G	50.0	IS	[38]
-penne §	durum wheat flour	47.0	4.0 º	capillary	10	G	50.0	Italy	[37]
-spaghetti *	semolina flour	47.0	n.a.	capillary	12	G	50.0	Iran	[41]
-spaghetti, dry *	durum wheat (*var. Svevo*) flour	48.0	4.0 º	n.a.	10	G	50.0	Italy	[42]
-penne §	durum wheat flour	50.0	7.0 º	capillary	10	G	50.0	Italy	[37]
-spaghetti §	durum wheat flour	50.0	9.0 º	capillary	10	G	50.0	Italy	[37]
-spaghetti §	durum wheat flour	51.0	9.0 º	capillary	10	G	50.0	Italy	[37]
-spaghetti, dry *	durum wheat (*var. Svevo*) flour	52.0	3.0 º	n.a.	10	G	50.0	Italy	[42]
-spaghetti, dry § (CT: 8 min)	durum wheat semolina	52.0	4.0 º	capillary	10	G	50.0	Italy	[43]
-spaghetti, dried at low temperature (55 °C) § (CT:10 min)	durum wheat flour	52.3	7.0 º	capillary	15	G	50.0	France	[44]
-pasta #	durum wheat semolina flour	52.5	8.4 º	capillary	15	G	50.0	Italy	[21]
-short penne §	durum wheat flour	53.0	5.0 º	capillary	10	G	50.0	Italy	[37]
-fusilli, dry # (CT: 10 min)	durum wheat flour	54.0	11.0 º	capillary	10	G	50.0	UK	[45]
-spaghetti #	durum wheat flour	54.9	n.a.	n.a.	10	WB	50.0	Italy	[46]
**Medium GI**									
-spaghetti * (CT: 15 min)	100% durum wheat semolina	58.0	6.8 º	capillary	10	WB	50.0	Sweden	[47]
-small penne §	durum wheat flour	59.0	11.0 º	capillary	10	G	50.0	Italy	[37]
-spaghetti *, infused	common wheat (*var. Nol*) flour	60.0	n.a.	capillary	12	G	50.0	Iran	[41]
-macaroni *	wheat flour	61.0	5.0 º	n.a.	10	WB	50.0	Canada	[48]
-fusilli * (CT: 10 min)	durum wheat semolina	61.0	9.0 º	n.a.	10	G	50.0	UK	[49]
-white spaghetti * and stored	white wheat flour	62.0	5.0 º	capillary	10	WB	50.0	Brasile	[50]
-spaghetti * and infused	semolina flour	63.0	n.a.	capillary	12	G	50.0	Iran	[41]
-white spaghetti and stored *	white wheat flour	64.0	7.0 º	capillary	10	WB	50.0	Brasile	[50]
-spaghetti *, infused	common wheat (*var. Nol*) flour	68.0	n.a.	capillary	12	G	50.0	Iran	[41]
-spaghetti *	white wheat flour	70.0	10.0 º	n.a.	12	WB	44.0	Australia	[40]
**High GI**									
-pasta *	wheat flour	72.6	n.a.	capillary	10	WB	n.a.	Indonesia	[18]
-spaghetti *	wheat refined flour	72.8	5.0 º	capillary	12	G	n.a.	Spain	[19]
-pasta, fresh # (CT: 20 min)	semolina flour	78.0	8.0 º	capillary	10	WB	50.0	Canada	[51]
-spaghetti *	white wheat flour	83.6	9.6 º	capillary	19	G	50.0	Canada	[33]
**Category n 2: 100% whole wheat**									
**Low GI**									
-spaghetti §	whole-meal durum wheat flour	35.0	3.0 º	capillary	10	G	50.0	Italy	[37]
-short penne §	whole-meal durum wheat flour	48.0	9.0 º	capillary	10	G	50.0	Italy	[37]
-spaghetti §	whole-meal durum wheat flour	53.0	10.0 º	capillary	10	G	50.0	Italy	[37]
-spaghetti §	whole-meal durum wheat flour	55.0	10.0 º	capillary	10	G	50.0	Italy	[37]
-fusilli, dry # (CT: 10 min)	whole wheat flour	55.0	8.0 º	capillary	10	G	50.0	UK	[45]
Medium GI									
-spaghetti *	whole wheat flour	65.0	n.a.	n.a.	10	WB	40.0	Canada	[40]
**Category n 3: other cereal-based products**									
**Low GI**									
-spaghetti, dried at high temperature (80 °C) *	emmer wheat flour (emmer genotype 399)	18.1	2.6 º	capillary	10	G	50.0	Italy	[36]
-spaghetti, dried at high temperature (80 °C) *	emmer wheat flour (emmer genotype 257)	30.5	4.7 º	capillary	10	G	50.0	Italy	[36]
-spaghetti #	Kamut^®^ (*T. polonicum*) flour	41.6	n.a.	n.a.	10	WB	50.0	Italy	[46]
**Medium GI**									
-spaghetti #	spelt (*T. dicoccum*) flour	56.5	n.a.	n.a.	10	WB	50.0	Italy	[46]
-pasta fresh # (CT: 5 min)	Celebrity barley cultivar (white pearled) flour	58.0	4.0 º	capillary	10	WB	50.0	Canada	[51]
-pasta fresh # (CT: 5 min)	AC Parkhill barley cultivar (white pearled) flour	64.0	4.0 º	capillary	10	WB	50.0	Canada	[51]
**High GI**									
-pasta fresh # (CT: 5 min)	Celebrity barley cultivar (whole grain) flour	71.0	6.0 º	capillary	10	WB	50.0	Canada	[51]
-pasta fresh # (CT: 5 min)	AC Parkhill barley cultivar (whole grain) flour	73.0	7.0 º	capillary	10	WB	50.0	Canada	[51]
**Category n 4: containing egg**									
**Low GI**									
-fettuccine *	egg pasta	47.0	n.a.	venous	14	G	46.0	NZ	[52]
-tagliatelle §	durum wheat flour, eggs	51.0	7.0 º	capillary	10	G	50.0	Italy	[37]
-lasagne, dry # (CT: 10 min)	egg pasta	53.0	9.0 º	capillary	10	G	50.0	UK	[45]
-tagliatelle *	egg pasta	54.0	5.0 º	capillary	10	G	50.0	UK	[53]
-tagliatelle, dry §	durum wheat flour, eggs	55.0	4.0 º	capillary	10	G	50.0	Italy	[37]
**Category n 5: gluten free**									
**Low GI**									
-penne, dry §	corn flour, millet flour, sugar cane syrup	48.1	n.a.	capillary	10	G	50.0	Italy	[34]
-spaghetti	rice and high amylose maize flour	51.0	5.0 º	n.a.	10	G	49.0	Australia	[40]
-pasta	rice flour	51.0	n.a.	n.a.	10	G	47.0	Australia	[40]
-fusilli, dry §	100% corn flour, water	54.4	n.a.	capillary	10	G	50.0	Italy	[34]
**Medium GI**									
-tagliatelle, fresh §	rice, corn and chickpea flour, eggs (20%), egg white, water	59.6	n.a.	capillary	10	G	50.0	Italy	[34]
-tortellini, fresh §	rice, corn and chickpea flour, eggs (20%), egg white, water, stuffed with pork meat	60.6	n.a.	capillary	10	G	50.0	Italy	[34]
-pasta macaroni, dry #	parboiled rice flour	61.0	n.a.	capillary	10	G	40.0	Italy	[54]
-pasta, macaroni dry #	parboiled rice flour	65.0	n.a.	capillary	10	G	40.0	Italy	[54]
-vermicelli *	finger millet flour, defatted soy, resistant maltodextrin	65.5	5.5 º	capillary	16	G	50.0	India	[55]
**High GI**									
-macaroni, dry #	rice flour	71.0	n.a.	capillary	10	G	40.0	Italy	[54]
-pasta *	corn flour	78.0	10.0 º	n.a.	10	G	42.0	Australia	[40]
**Category n 6: containing legumes**									
**Low GI**									
-pasta #	60% grass pea flour, 40% chickpea flour	20.0	7.6 º	capillary	15	G	50.0	Italy	[21]
-pasta #	100% red lentil flour	22.3	6.9 º	capillary	15	G	50.0	Italy	[21]
-pasta #	100% pea flour	23.3	6.7 º	capillary	15	G	50.0	Italy	[21]
-spaghetti, dried at low temperature (55 °C) § (CT: 10.5 min)	35% faba bean flour, durum wheat semolina	41.9	5.7 º	capillary	15	G	50.0	France	[44]
-spaghetti	soy flour	47.0	7.4 º	capillary	10	G	25.0	Australia	[32]
-spaghetti, dried at high temperature (90 °C) § (CT: 13.5 min)	35% faba bean flour, durum wheat semolina	49.4	6.8 º	capillary	15	G	50.0	France	[44]
-macaroni *	50% red lentil flour	55.0	8.0 º	n.a.	10	WB	50.0	Canada	[48]
**Medium GI**									
-spaghetti * (CT: 10 min)	75% durum wheat flour, 25% chickpea flour	58.9	6.0 º	capillary	12	G	n.a.	Spain	[19]
**High GI**									
-spaghetti *	30% whole yellow pea flour, white durum wheat flour	93.3	9.4 º	capillary	19	G	50.0	Canada	[33]
**Category n 7: noodles and vermicelli**									
**Low GI**									
-noodles, dry *	wheat flour	46.0	5.8 º	venous	10	G	50.0	China	[56]
-noodle, dried	wheat	46.0	2.0 º	n.a.	10	G	42.0	China	[40]
-noodles, instant ‘two-minute’	n.a.	48.0	n.a.	venous	15	G	26.0	NZ	[52]
-noodles, instant, all flavors	n.a.	52.0	5.0 º	n.a.	10	G	22.0	Australia	[40]
**Medium GI**									
-Jianxi vermicelli * (CT: 8 min)	rice flour	56.0	7.0 º	capillary	10	G	50.0	HK	[57]
-Sau tao Beijing noodles * (CT: 3 min)	wheat flour, salt, tapioca starch	61.0	5.0 º	capillary	10	G	50.0	HK	[57]
-noodles, reheated (CT: 5 min)	udon pasta, plain	62.0	8.0 º	n.a.	10	G	48.0	Australia	[40]
-Sau tao chicken-flavored Sichuan spicy noodles * (CT: 3 min)	wheat flour, salt	65.0	4.0 º	capillary	10	G	50.0	HK	[57]
-Taiwan vermicelli * (CT: 2 min)	rice, maize starch	68.0	12.0 º	capillary	10	G	50.0	HK	[57]
**Category n 8: containing vegetable or algae**									
**Low GI**									
-small farfalle §	durum wheat flour, carrot and pumpkin pulps	44.0	5.0 º	capillary	10	G	50.0	Italy	[37]
-pasta, dry *	wheat flour, algae (*eucheuma cottonii*) flour (21%), eggs, cooking oil	44.4	n.a.	capillary	10	WB	N/A	Indonesia	[18]
-small pipe §	durum wheat flour, tomato and carrot pulps	47.0	7.0 º	capillary	10	G	50.0	Italy	[37]
-small penne §	durum wheat flour, zucchini and spinach pulps	48.0	5.0 º	capillary	10	G	50.0	Italy	[37]
**Medium GI**									
-pasta, dry *	wheat flour, algae (*eucheuma cottonii*) flour (14%), eggs, cooking oil	56.3	n.a.	capillary	10	WB	n.a.	Indonesia	[18]
-pasta, dry *	wheat flour, algae (*eucheuma cottonii*) flour (7%), eggs, cooking oil	66.4	n.a.	capillary	10	WB	n.a.	Indonesia	[18]
**Category n 9: containing other ingredients**									
**Low GI**									
-pasta *	protein enriched	28.0	1.0 º	n.a.	10	G	49.0	Australia	[40]
-spaghetti, dry § (CT: 8.5 min)	85% durum wheat semolina, 15% Barley Balance^®^	33.0	5.0 º	capillary	10	G	50.0	Italy	[43]
-spaghetti, dry § (CT: 8 min)	85% durum wheat semolina, 7.5% Barley Balance^®^, 7.5% psyllium seed husk	35.0	3.0 º	capillary	10	G	50.0	Italy	[43]
-spaghetti, dry *	durum wheat (*var Svevo*, line SBEIIa) flour, 58% amylose, 7.36% RS	38.0	3.0 º	n.a.	10	G	50.0	Italy	[42]
-spaghetti, dry *	durum wheat (*var Svevo* line SSIIa) flour, 44% amylose, 2.06% RS	49.0	3.0 º	n.a.	10	G	50.0	Italy	[42]
**Category n 10: stuffed**									
**Medium GI**									
-ravioli, fresh §	durum wheat flour, stuffed with calf meat	58.0	7.0 º	capillary	10	G	50.0	Italy	[37]

GI = glycemic index; º = data distribution is expressed as standard error of mean (SEM); ** = data distribution is expressed as standard deviation (SD); G = glucose solution; WB = white bread; Av. CHO = available carbohydrates; n.a. = not available; UK = United Kingdom; NZ = New Zealand; HK: Hong Kong; IS = interlaboratory study: the study was performed in Canada, Italia, Australia, Sweden, New Zealand, West Indies and South Africa; GF = gluten free; § = boiled in salted water; # = boiled in unsalted water; * = boiled in water; CT = cooking time; RS = resistant starch; Var = variety.
